# Diagnostic moléculaire du Cytomégalovirus (CMV), de l’herpès virus humain de type 6 (HHV6) et d’Epstein-Barr virus (EBV) par PCR en temps réel chez les femmes enceintes VIH séropositives et séronégatives à Ouagadougou, Burkina Faso

**DOI:** 10.11604/pamj.2016.24.223.9406

**Published:** 2016-07-12

**Authors:** Alice Rogomenoma Ouedraogo, Madeleine Kabre, Cyrille Bisseye, Théodora Mahoukèdè Zohoncon, Maleki Asshi, Serge Théophile Soubeiga, Birama Diarra, Lassina Traore, Florencia Wendkuuni Djigma, Djénéba Ouermi, Virginio Pietra, Nicolas Barro, Jacques Simpore

**Affiliations:** 1 Centre de Recherche Biomoléculaire Pietro Annigoni (CERBA), LABIOGENE UFR/SVT, Université de Ouagadougou BP 364 Ouagadougou, Burkina Faso; 2 Laboratoire de Biologie Moléculaire et Cellulaire (LABMC), Université des Sciences et Techniques de Masuku (USTM), Franceville, Gabon; 3 Laboratoire de Biologie Moléculaire, d’Epidémiologie et de Surveillance des Bactéries et virus Transmissibles par les Aliments (LaBESTA), UFR/SVT, Université de Ouagadougou

**Keywords:** HHVs, VIH, PCR multiplex en temps réel, femme enceinte, Burkina Faso, HHVs, HIV, multiplex real time PCR, pregnant woman, Burkina Faso

## Abstract

**Introduction:**

Les herpès virus EBV, CMV et HHV-6 sont des virus qui évoluent sous le modèle pandémique et sont responsables d’infections congénitales pouvant provoquer des séquelles graves chez les nouveau-nés. L’objectif de cette étude était de déterminer les prévalences de CMV, EBV et HHV-6 chez les femmes enceintes VIH(+) et VIH(-) à Ouagadougou.

**Méthodes:**

Dans cette étude 200 échantillons de plasma sanguin de femmes enceintes dont 100 femmes VIH(+) et 100 femmes VIH(-) ont été diagnostiqués par PCR multiplex en temps réel pour les trois infections (EBV, CMV et HHV-6).

**Résultats:**

Sur l’ensemble des 200 échantillons analysés, 18 (9,0%) étaient positifs à au moins un des trois virus, 12 (6,0%) étaient positifs au EBV, 13 (6,5%) au CMV et 12 (6,0%) positifs au HHV-6. Parmi les 18 cas d’infections, nous avons trouvé 10 cas (55,6%) de coïnfections dont 90,0% (9/10) d’infection multiple EBV/CMV/HHV6 et 10,0% de coinfection EBV/HHV6. Le taux d’infection HHVs était plus élevé chez les femmes VIH(-) que celles VIH(+) (12,0% versus 6,0%). Parmi les VIH(+), la PCR a révélé 7,1% (soit 6/85) d’infection HHVs chez celles qui n’étaient pas sous ARV contre 0% chez celles sous ARV.

**Conclusion:**

Les herpès virus sont fréquents chez les femmes enceintes au Burkina Faso et pourraient constituer une menace chez ces dernières à cause des complications et des risques d’infection pour le nouveau-né.

## Abstract (en)

**Introduction:**

Herpesvirus EBV, CMV and HHV-6 are viruses that evolve based on pandemic modeling and are responsible for congenital infections causing severe sequelae in infants. This study aims to determine the prevalence of CMV, EBV and HHV-6 among HIV (+) and HIV (-) pregnant women in Ouagadougou.

**Methods:**

In this study 200 blood plasma samples taken from pregnant women, of whom 100 with HIV(+) and 100 with HIV(-), were analyzed using multiplex real-time PCR which detected three infections (EBV, CMV and HHV-6).

**Results:**

Out of the 200 samples tested, 18(9.0%) were positive for at least one of the three viruses, 12(6.0%) were positive for EBV, 13(6.5%) were positive for CMV and 12(6.0%) were positive for HHV-6. Among the 18 cases with infections, 10 cases (55.6%) had co-infections of whom 90.0% (9/10) with multiple EBV/CMV/HHV6 infection and 10.0% with EBV/HHV6 co-infection. HHVs infection rate was higher among HIV (-) pregnant women than among HIV (+) pregnant women (12.0% versus 6.0%). Among HIV (+) pregnant women, PCR showed 7.1% (6/85) of HHVs infection in patients who were not treated with ARV against 0% in those treated with ARVs.

**Conclusion:**

Herpes virus infections are a common condition in pregnant women in Burkina Faso. They may represent a real threat to pregnant women because of complications and risks of infection in infants.

## Introduction

Les herpès virus humains (HHVs) font partie des virus à ADN les plus répandus, responsables de diverses pathologies chez les humains [1] notamment de la mononucléose infectieuse, de l’exanthème subit et de nombreux cancers tels que le cancer de Burkitt, du nasopharynx, du colon, et le cancer du cerveau [2, 3]. Les infections au Cytomégalovirus (CMV), au Epstein Barr virus (EBV) et à l’herpès virus 6 (HHV-6) sont généralement asymptomatiques chez les personnes immunocompétentes mais graves chez l’immunodéprimé [4] et la femme enceinte [5]. Des études antérieures ont révélé des prévalences très diversifiées de ces virus chez les femmes enceintes à travers le monde [6–8]. Les études antérieures se rapportaient principalement au CMV avec une prévalence de 72,1% en Iran [9], 30,0% en Israël [10] et 12,0% en Egypte [11]. Le risque de transmission de la mère à l’enfant variait de 40% -50% en Philadelphie [12], de 24% à 75% aux USA [13] et de 30,8% en Italie [12]. La coïnfection HHVs et virus de l’immunodéficience humaine (VIH) est un facteur de risque de la persistance de l’infection à HHVs en Afrique [1]. En 2005, environ 4,3% des femmes de l’Afrique subsaharienne présentaient une infection à VIH [14]. Alors que les herpès virus constituent un véritable problème de santé publique pour les femmes enceintes et les nouveau-nés, très peu de données existent sur la prévalence de ces virus chez les femmes enceintes en Afrique et plus particulièrement au Burkina Faso. Cependant, la capacité à identifier de manière fiable leur primo-infection pendant la grossesse, permet de réduire le taux d’infection et de mortalité infantile. L’objectif de cette étude était de diagnostiquer par PCR multiplex en temps réel le CMV, EBV et l’ HHV6 chez les femmes enceintes fréquentant le service PTME du centre médicale Saint Camille de Ouagadougou.

## Méthodes

**Cadre et population d’étude:** la présente étude s’est déroulée au centre Médical Saint Camille à Ouagadougou, Burkina Faso. De Janvier à Juin 2013, deux cent (200) femmes enceintes sans distinction d’âge ni de stade de grossesse ont été incluses dans cette étude dont cent (100) femmes VIH séropositives et cent (100) femmes VIH séronégatives. Certaines d’entre elles étaient déjà incluses dans le programme de Prévention de la Transmission Mère-Enfant (PTME) ou référées au CMSC avant accouchement et avaient préalablement donné leur consentement libre et éclairé après avoir reçu d’amples explications sur l’intérêt de l’étude à laquelle elles sont invitées à participer.

**Collecte des échantillons:** les femmes enceintes ont d’abord été dépistée au VIH au service PTME. Les échantillons de sang prélevés ont été centrifugés et les plasmas ont été conservés à - 20°C.

**Extraction et amplification de l’ADN viral (EBV, CMV, HHV6):** l’ADN viral a été extrait à partir du plasma à l’aide du kit « DNA-Sorb-B » (SACACE biotechnologies ®, Italie). L’amplification a été réalisée en utilisant le kit *«Multiplex Real Time PCR Kit for quantitative detection and differentiation of Cytomegalovirus, Epstein Barr virus and Human Herpes 6 virus, SACACE biotechnologies ®, Italie»* à l’aide de l’appareil PCR, SaCycler-96 Real Time PCR v.7.3 (Sacace Biotechnologies). Le volume réactionnel était de 25µl dont 10µl d’ADN. Le programme d’amplification sur SaCycler-96 pour la PCR multiplex en temps réel des HHVs était le suivant: 1 cycle de 95°C pendant 15 minutes, 5cycles composés de 95°C pendant 05s, 60°C pendant 20s, 72°C pendant 15s; et 40 cycles composés de 95°C pendant 05s, 60°C pendant 30s et 72°C pendant 15s.

**Analyses statistiques**: les données ont été traitées et analysées à l’aide des logiciels SPSS 17.0 (Standard Statistical Package for Social Sciences version 17.0) et Epi Info 3.5.1. Le test de Chi carré a été utilisé pour les comparaisons. La différence a été significative pour p < 0,05.

**Considérations éthiques**: le consentement libre et éclairé des sujets a été obtenu avant la collecte du sang. L’étude a été approuvée par le comité d’éthique institutionnelle du CERBA/LABIOGENE.

## Résultats

**Caractéristiques sociodémographiques des femmes enceintes dépistées aux HHVs:** l’âge moyen des femmes enceintes était de 28,18 ± 5,95 ans. Parmi les femmes enceintes VIH séropositives, l’âge moyen était de 30,04 ± 5,54 ans et celui des VIH séronégatives était de 26,31 ± 5,79 ans (Tableau 1). Parmi les 100 femmes VIH séropositives, 7,50% (15/200) étaient sous ARV dont 6% (12/200) sous prophylaxie ARV et 1,5% (03/200) sous trithérapie.

**Tableau 1:** Caractéristiques sociodémographiques de ces femmes enceintes

	Femmes VIH-n (%)	Femmes VIH+n (%)	Totaln (%)
**Age**			
≤ 20	20(20,00)	4(4,00)	24(12,00)
] 20-30]	57(57,00)	52(52,00)	109(54,50)
> 30	23(23,00)	44(44,00)	67(33,50)
**Profession**			
Salariées	8(8,00)	10(10,00)	18(9,00)
Ménagères	43(43,00)	64(64,00)	107(53,50)
Elèves/Etudiantes	16(16,00)	5(5,00)	21(10,50)
Secteur informel	33(33,00)	21(21,00)	54(27,00)
**Protocole ARV**			
Prophylaxie	-	12(12,00)	12(6,00)
Traitement	-	3(3,00)	3(1,50)

**Prévalence des infections HHVs (EBV, CMV, HHV6) dans la population d’étude:** sur les 200 échantillons analysés, 9,00% (18/200) étaient positifs à au moins un des trois herpès virus (EBV=2, CMV=4, HHV6= 2, EBV/HHV6=1, EBV/CMV/HHV6=9). L’infection la plus fréquente était celle liée au CMV (6,50% soit 13/200) suivie des infections au EBV (6,00% soit 12/200) et au HHV6 (6,00% soit 12/200) (Tableau 2). La coïnfection EBV/CMV/HHV6 était de 90% (9/10) contre 10%(1/10) d’EBV/HHV6. Par ailleurs, les coïnfections EBV/CMV ou CMV/ HHV6 n’ont pas été détecté chez les femmes enceintes de notre étude.

**Tableau 2:** Infections HHVs en fonction des caractéristiques sociodémographiques des femmes enceintes et impact du traitement antirétroviral

	HHVsn (%)	EBV^+^n (%)	CMV^+^n (%)	HHV6^+^n (%)	Taux coinfection n (%)	Total
**Caractéristiques**						
**Age (ans)**						
≤ 20	3 (12,5)	2(8,33)	1(4,17)	1(4,17)	1(4,17)	24
] 20-30]	7 (6,42)	7(6,42)	6(5,50)	6(5,50)	6(6,50)	109
> 30	8(11,94)	3(4,48)	6(8,96)	5(7,46)	3(4,48)	67
**Terme grossesse**						
1^er^ trimestre	4 (5,63)	4 (5,63)	3 (4,23)	4 (5,63)	4 (5,63)	71
2^ème^ trimestre	11(12,36)	6 (6,74)	7 (7,87)	6 (6,74)	4 (4,49)	89
3^ème^ trimestre	3 (7,5)	2 (5)	3 (7,5)	2 (5)	2 (5)	40
**Avortement**						
Oui	3(12,5)	1 (4,17)	2 (8,33)	2 (8,33)	1 (4,17)	24
Non	15(8,52)	11(6,25)	11(6,25)	10(5,68)	9 (5,11)	176
**Sérologie VIH**						
VIH (-)	12 (12)	7(7)	8(8)	8(8)	6 (6)	100
VIH (+)	6 (6)	5(5)	5(5)	4(4)	4 (4)	100
**ARV**						
Sans ARV	6 (7,06)	5(5,88)	5(5,88)	4(4,71)	10(11,76)	85

**Infections HHVs en fonction des caractéristiques sociodémographiques des femmes enceintes et impact du traitement antirétroviral:** la prévalence HHVs était de 12,50% pour les femmes ayant moins de 20 ans, 6,42% pour celles ayant un âge compris entre 20 et 30 ans, 11,94% pour un âge supérieur à 30 ans. La forte fréquence HHVs (61,11%) était au 2^ème^ trimestre de grossesse. Les infections à HHVs sont beaucoup plus accentuées chez les femmes enceintes n’ayant pas d’antécédents d’avortement que chez celles qui en avaient mais cette différence n’était pas statistiquement significative (Tableau 2). Douze (12,00) % de femmes VIH(-) présentait une infection HHVs contre 6,00% chez les VIH(+). Les taux de coïnfections HHVs (CMV, EBV, HHV6) étaient respectivement de 4,17%, 6,50% et 4,48%. Dans la population de femmes enceintes VIH séropositives sous thérapie ARV, le taux d’infection aux HHVs était de 7,06% (soit 6,00/85) contre 0% chez celles soumises à une thérapie ARV (Tableau 2).

## Discussion

L’objectif principal de cette étude était de diagnostiquer par la PCR multiplexe en temps réel le CMV, EBV et HHV6 chez les femmes enceintes fréquentant le service PTME du centre médicale Saint Camille de Ouagadougou. Les prévalences de 6,00%, 6,50% et 6,00% respectivement pour EBV, CMV et HHV6 observées dans la population générale de femmes enceintes sont supérieures à celles de 0,10%, 0,80%, 1,00% rapportées en Italie [7]. La discordance des résultats HHVs peut s’expliquer non seulement par la nature du fluide biologique utilisé mais aussi par les stades d’infection (primo-infection, réactivation, réinfection), l’état immunologique de la femme enceinte, la sensibilité et spécificité des tests (PCR multiplex en temps réel et la PCR quantitative en temps réel). En effet, la virémie serait plus élevée dans l’urine maternelle [6] et le liquide amniotique [7] que dans le plasma sanguin. La prévalence de 6,5% de l’infection par CMV dans la population générale de femmes enceintes est comparable aux prévalences de 4,5% et 10,11% rapportées au Koweït [6] à travers la PCR nichée appliquée respectivement au sang du cordon et à l’urine maternelle. A l’inverse, cette prévalence du CMV est inférieure à celles de 12,00%, 18,18%, 30,00%, 30,16% rapportées respectivement en Egypte [11] à travers des tests sérologiques de patientes ASR (avortement spontané récurrent), en Suisse [15] par PCR (amniocentèse et sang fœtal), en Israël [10] grâce à la PCR effectué sur des grossesses gémellaires à travers le liquide amniotique et la culture du CMV après naissance, en Allemagne [16] par des tests sérologiques (sang fœtal, liquide amniotique). La prévalence de 6,00% de l’infection à EBV est inférieure à celle de 98% trouvée en Caroline du nord (USA) [8] à travers des tests sérologiques.

La prévalence 6,00% de HHV6 est supérieure à celle de 1,00% rapportée en Italie [7] et 1,00% aux Etats Unis [17]. Cependant, cette prévalence dépend non seulement de sa localisation mais aussi du terme de grossesse. En effet, à New York, de la première moitié à la deuxième moitié de grossesse, cette prévalence était respectivement de 25,80% et 22,20% dans les cellules mononucléaires de sang périphériques (PBMC), 7,50% et 7,10% dans les sécrétions cervicales, 9,10% dans le placenta [17]. La fréquence HHVs (61,11%) détectée au 2^ème^ trimestre de grossesse est conforme à la prévalence de 2,20% d’acides nucléiques viraux de HHV6 détectés dans le liquide amniotique au cours du 2^ème^ trimestre de grossesse en Italie [7] et 35% de réactivation d’EBV rapporté en Caroline du Nord [8]. Cette prévalence pourrait constituer un haut risque de transmission des herpès virus au fœtus. L’accentuation des infections HHVs chez les femmes enceintes n’ayant pas d’antécédents d’avortement pourrait s’expliquer par le fait que certains herpès virus sont responsables de certains cas d’avortement et pourraient de ce fait conférer à l’organisme maternel une certaine immunité. En Afrique, la coïnfection HHVs et virus de l’immunodéficience humaine (VIH) est un facteur de risque additionnel [1]. Cependant, dans notre échantillon la prévalence de l’infection à au moins un des trois HHVs était plus élevée chez les femmes enceintes VIH séronégatives que chez les VIH séropositives. Cependant cette différence n’était pas significative (P= 0,138). L’absence d’infection HHVs parmi les femmes enceintes VIH séropositives soumises à la thérapie ARV de notre étude, corrobore avec les résultats trouvés en Italie [18]. En effet, la thérapie antirétrovirale semble augmenter le taux de cellules T CD4, empêchant ainsi la réactivation des herpès virus. Cela est conforme aux résultats rapporté au Burkina Faso [19] sur le HHV8/VIH et en Alabama aux USA [20] sur l’impact de la trithérapie HAART sur la prévalence du CMV. La principale limite de cette étude est la taille non représentative de la population féminine d’Ouagadougou. Cependant, cette étude pilote à une importance épidémiologique et clinique, car elle permet d’estimer la prévalence du CMV, d’EBV et de HHV6 chez les femmes enceintes à Ouagadougou. En outre elle indique que la prévalence des HHVs au Burkina Faso est différente de celle des autres régions. Cette étude peut avoir un réel impact sur le choix de la politique de lutte non seulement contre les infections aux HHVs ainsi qu’au VIH, mais aussi contre les risques d’infections congénitales et de développement de certains cancers associés.

## Conclusion

Nous avons montré dans cette étude une prédominance des infections au CMV, EBV et HHV6 dans une cohorte de femmes enceintes VIH séropositives et VIH séronégatives. La vulnérabilité des femmes enceintes aux HHVs ainsi que les risques d’infections congénitales ont été montré.

### Etat des connaissances actuelles sur le sujet

- Les femmes VIH+ présentent plus d’infections HHVs;
- Les femmes VIH- présentent moins d’infections HHVs;
- La prévalence des HHVs varie en fonction des régions.

### Contribution de notre étude à la connaissance

- Les ARV réduisent le risque de coinfection HHVs/VIH;
- Les femmes enceintes VIH+ peuvent posséder certains facteurs permettant de réduire le risque d’infection HHvs au profit du VIH.
